# Psychometric Properties of the Greek Haem-A-QoL for Measuring Quality of Life in Greek Haemophilia Patients

**DOI:** 10.1155/2014/968081

**Published:** 2014-05-06

**Authors:** Agoritsa Varaklioti, Nick Kontodimopoulos, Olga Katsarou, Dimitris Niakas

**Affiliations:** ^1^Blood Center and National Centre for Congenital Bleeding Disorders, Laiko General Hospital, Ag. Thoma 17, 11527 Athens, Greece; ^2^Hellenic Open University, Parodos Aristotelous 18, 26335 Patra, Greece

## Abstract

*Background and Objectives*. Health Related Quality of Life (HRQoL) is an important health outcome measure in haemophilia. The aim of this study was to assess the psychometric properties of the Greek version of Haem-A-QoL, a disease-specific questionnaire for haemophiliacs. * Methods*. Haem-A-QoL and SF-36 were administered to 118 adult haemophilia patients. Hypothesized scale structure, internal consistency (Cronbach's **α**), and test-retest reliability, as well as various types of construct validity were evaluated. * Results*. Scale structure of Haem-A-QoL was confirmed, with good item convergence (87%) and discrimination (80.6%) rates. Cronbach's **α** was >0.70 for all but one dimension (dealing) and test-retest reliability was significantly high. The strength of Spearman's correlations between Haem-A-QoL and SF-36 scales ranged from 0.25 to 0.75 (*P* < 0.01). Multiple stepwise linear regression analysis revealed that all but one Haem-A-QoL dimensions were important predictors of SF-36 scales. Known-groups comparisons yielded consistent support of the instruments' construct validity and significant relationships were identified for age, educational level, haemophilia type, disease severity, and viral infections. * Conclusion*. Overall, the psychometric properties of the Greek version of Haem-A-QoL, resulting from this first time administration of the instrument to Greek adult haemophiliacs, confirmed it as a reliable and valid questionnaire for assessing haemophilia-specific HRQoL in Greece.

## 1. Introduction

Haemophilia is a rare congenital bleeding disorder, characterized by posttraumatic and spontaneous bleeds, mainly in joints and muscles, leading to joint degradation, painful arthritis, and arthropathy. Deficiency of coagulation factors VIII and IX accounts for haemophilia A and haemophilia B, respectively. Severity of the disease is associated with the quantity levels of factors VIII and IX in the patients' plasma; haemophilia is described as severe, moderate, and mild, when the residual plasma activity of the clotting factor is <1%, between 1% and 5%, and between 5% and 40%, respectively [1–3]. Both haemophilia A and haemophilia B are X-linked genetic disorders and therefore occur almost exclusively in males. It is estimated that the number of haemophilia patients worldwide is approximately 400,000. In Greece 912 patients are estimated to suffer from haemophilia A or B, 716 of which are adult patients [4].

Standard treatment is based on the intravenous replacement therapy with concentrates of coagulation factors either when bleeding occurs (on demand therapy) or preventively [1]. Traditional outcome measures of haemophilia care are restricted to clinical outcomes, such as arthropathy-associated pain, number of joint bleeds, and loss of motion range.

Health-related quality of life (HRQoL) is increasingly recognized as a significant component of modern haemophilia management and as an important health outcome measure in haemophilia, which may help in improving treatment strategies. Several studies in the literature assess HRQoL in haemophilia adult patients, using mainly generic instruments, like SF-36, SF-12, and EQ-5D [5–7]. Lately, a few disease-specific questionnaires for haemophilia patients have been developed and validated. These include Haemo-QoL, CHO-KLAT, and QoL questionnaire for Young patients, which were designed for children [8–11], while Hemo-QoL-A, Hemophilia-QoL, Hemolatin-QoL, and Haem-A-QoL apply to adult patients [12–17]. The latter was developed and pilot-tested using patient-based focus groups (*n* = 32) and expert groups organized with physicians and nurses initially [18]. It was validated in 233 Italian adult haemophilia patients [19] and has been linguistically validated in more than 30 languages, including Greek [20, 21].

The aim of the present study was the validation of the Greek version of Haem-A-QoL, a disease-specific questionnaire, and the evaluation of its psychometric properties, with regard to reliability (internal consistency and test-retest) and construct validity. To the best of our knowledge, this is the first study in Greek adult haemophilia patients with a disease-specific instrument. This validated Greek version of Haem-A-QoL questionnaire can be subsequently utilized in future studies monitoring HRQoL in adult haemophilia patients in Greece.

## 2. Methods

### 2.1. Instruments

Haem-A-QoL was designed for adult patients with haemophilia. It consists of 46 items comprising 10 dimensions (“physical health,” “feelings,” “view,” “sport and leisure time,” “work and school,” “dealing,” “treatment,” “future,” “family planning,” and “relationships/partners”) and a scale representing total score [15, 18, 22, 23]. Scoring is performed by transforming the scores achieved in each dimension, as well as the total score, on scales ranging from 0 to 100, with 0 representing the best and 100 the worst HRQoL [24]. The Greek version of the Haem-A-QoL questionnaire was used in this study with the expressed permission of the Haemo-QoL group.

The Short Form Health Survey (SF-36) is a self-administered generic HRQoL questionnaire for adults [25], allowing the comparison of a specific disease such as haemophilia with norm data of the general population related to gender and age groups. It consists of 36 items pertaining to eight dimensions of HRQoL (PF: physical functioning; RF: role physical; BP: bodily pain; GH: general health perception; VT: vitality; SF: social functioning; RE: role emotional functioning; and MH: mental health). Each of the eight domains can be transformed into scores ranging from 0 (worst quality of life) to 100 (best quality of life); two summary scores can be calculated, for physical component score (PCS) and mental component score (MCS), respectively. The SF-36 has been translated, psychometrically tested, and normed in over 30 countries and is available in most European languages. The validated Greek version [26] of the generic SF-36 Health Survey was used as the “gold standard” for HRQoL assessment in this study.

### 2.2. Patients and Data Collection

The study was conducted in the National Centre for Congenital Bleeding Disorders in Laiko General Hospital of Athens. The Hospital's Review Board granted ethical approval. The data were collected between September 2011 and March 2012 and the sample consisted of adult patients with haemophilia A and haemophilia B. The survey included the SF-36 and the Greek version of the Haem-A-QoL questionnaires as well as sociodemographic questions, which were administered for self-completion. All the necessary clinical data were obtained from the patients' medical records. Completion time was approximately 30 min, and 118 out of 122 visiting the facility during the study period agreed to participate (96.72% response rate). During the study period, 30 patients returned within 15 days after their initial visit and were asked to complete the two questionnaires again. 23 out of 30 agreed and the response rate of repeatedly completed questionnaires was 76.7%.

### 2.3. Data Analysis

Percentages of ceiling and floor scores were calculated as an indication of the instrument ability to detect changes over time. Scale internal consistency reliability was assessed using Cronbach's *α* and the 0.70 standard for group-level comparisons was adopted [27]. Test-retest reliability was assessed using the correlation coefficient over a period of about 2 weeks. Item-internal consistency, which is substantial when correlation between an item and its hypothesized scale (corrected for overlap) is >0.40, and item discriminant validity, which is successful when correlation between an item and its own scale is significantly higher (>2 standard errors) than with other scales, were used to examine the hypothesized scale structure.

Spearman's correlations between Haem-A-QoL and SF-36 scales were used to assess convergent construct validity. Based on the literature, scales measuring similar HRQoL dimensions were hypothesized to be strongly correlated (>0.50) [28]. Construct validity was also examined using the interscale correlations between Haem-A-QoL and SF-36, conceptually related scales would substantially correlate (>0.40) and scales with less in common would show lower correlations. Each SF-36 scale was separately regressed against the Haem-A-QoL dimensions in order to identify aspects of the disease-specific instrument more closely linked to general HRQoL [29]. Known-groups' validity was assessed by testing Haem-A-QoL's ability to discriminate between groups of patients known to differ by sociodemographic and clinical characteristics. Age group, educational level, employment, and family status, as well as several clinical characteristics were used as testing criteria and differences were examined with *t*-test and ANOVA, Kruskal Wallis, and Mann Whitney tests. Multivariate OLS regression analysis identified independent sociodemographic, clinical, and treatment-related variables that affect HRQoL. All analyses were performed using SPSS software, version 19.0 (SPSS Inc, Chicago IL).

## 3. Results

The sociodemographic and clinical characteristics of the participating patients are shown in Table 1. The patients' age ranged between 18 and 75 years old, with a median age of 39 and mean age 39.6 (±12.9). Haemophilia is an X-linked genetic disease; therefore the majority of the patient sample was men (99.2%). The only woman in the sample, who was a carrier of the haemophilia gene, had low levels of factor VIII and similar to mild haemophilia patient symptoms. The majority of the patients were single (61%), worked as employees (32.2%), had graduated from high school (57.6%), and lived in the greater area of Attica (57.6%). The predominant form of haemophilia was A (78%) and 63.6% of the individuals had severe haemophilia. Most of the patients suffered from hemarthrosis (78.0%), muscle hematomas (89.8%), and other types of bleeding (74.6%). Regarding viral infections, 84 patients (71.2%) were HCV positive and 33 patients (28.0%) were HIV positive. Concerning replacement treatment, 14.4% of the patients received constant and 9.3% transient prophylaxis and recombinant factor concentrates were administered to 94.9% of the patients (Table 1).

Data on central tendency, variability, and reliability of Haem-A-QoL are depicted in Table 2. No noteworthy floor or ceiling effects were observed in all the dimensions of the Haem-A-QoL instrument. Only one dimension (relationship/partnership) suffered from a marginally high floor effect (40.7%). Throughout the instrument, only one scale (dealing) did not meet the 0.70 internal consistency criterion (0.585). Additionally, Haem-A-QoL demonstrated increased test-retest reliability with a significantly high correlation coefficient (>0.87) in all the scales.

Significantly higher item-scale correlations between items and their hypothesized scales than with competing scales were observed (Table 3). The 0.40 item-scale correlation criterion was satisfied, confirming item convergence in 40/46 (87.0%) tests for Haem-A-QoL. Accordingly, item discrimination was successful in 341/423 (80.61%) of the respective tests. The most obvious scaling failures occurred in the treatment and relationship/partnership scales.

Convergent construct validity was tested by examining conceptually related correlations between the two instruments used, based on findings from the literature. All correlations between generic (SF-36) and disease-targeted (Haem-A-QoL) scales, shown in Table 4, were negative, due to the reversed scoring between the two instruments, and strong except for one scale (dealing). Six out of nine hypothesized strong correlations (>0.50) between pairs of Haem-A-QoL and SF-36 scales measuring similar dimensions were confirmed (PF, RP, BP, and PCS of SF-36 with Physical Health of Haem-A-QoL, MH, and MCS of SF-36 with feelings of Haem-A-QoL) with correlations ranging from 0.53 to 0.75 and three correlations were borderline (feelings, family planning, and relationship/partnership with *P* = 0.43, 0.47, and 0.45, resp.) (Table 4). Noteworthy, strong correlations were observed between almost all dimensions of the Haem-A-QoL with SF-36 scales (*P* > 0.50). All hypothesized strong correlations were statistically significant (*P* < 0.001).

Multiple stepwise linear regression analysis identified those Haem-A-QoL dimensions most closely associated with specific domains of HRQoL, as represented by SF-36 scales. In Table 5, only the significant predictors are demonstrated. All Haem-A-QoL dimensions were significantly related to at least SF-36 scale. Only one dimension (dealing) was not a significant predictor of any SF-36 scale. Explanatory power was satisfactory for most models, with the general health (64.6%) and bodily pain (58.3%) models demonstrating the highest (Table 5).

The ability of the instrument to distinguish between known groups was confirmed with statistically significant correlations between several Haem-A-QoL scales and four sociodemographic variables, that is, age, family status, educational level, and employment, and six disease-specific variables, namely, haemophilia type, disease severity, muscle hematomas, prophylaxis treatment, HCV, and HIV infection (Table 6).

Known-groups validity was confirmed, by comparing mean scale scores across groups known to differ, using sociodemographic and clinical characteristics as the differing criteria. As shown in Table 7, significantly higher scores (lower HRQoL) were observed with increasing age in most of the dimensions (physical health, view, sports/leisure, work/school, treatment, relationships, and total score). Differences were also observed in three dimensions, namely, physical health, work/school and total score, with educational level as the criterion, with more years of education associated with better-reported health (*data not shown*). Accordingly, higher scores (lower HRQoL) were observed in patients with haemophilia A in all the Haem-A-QoL dimensions, and in six of them this correlation was also statistically significant. Furthermore, as expected, haemophilia patients with either HCV or HIV infection presented lower HRQoL, as revealed by the higher scores in all but one (dealing) Haem-A-QoL dimension (Table 7).

The significance of the aforementioned relationships between sociodemographic and clinical variables and Haem-A-QoL dimensions was confirmed with multivariate OLS regressions (Table 8). In all but three dimensions (feelings, dealing, and treatment), viral infection (HCV or HIV) was identified as a significant predictor of decreased HRQoL, with the regression models explaining between 9% and 20% of the variance. Another clinical variable, prophylaxis treatment, was also recognized as a significant predictor of diminished HRQoL in six dimensions, with the models explaining between 3.2% and 20% of the variance. Moreover, type of haemophilia was also identified as an important predictor of HRQoL in 5 scales, with the regression models explaining between 3.7% and 18.1% of the variance (Table 8). Among the sociodemographic variables, age and educational level were the most pronounced predicting factors of reduced perceived health in four and six Haem-A-QoL scales, respectively, and the regression models explaining between 3.3% and 13.5% and 3.3 and 18.2% of the variance (Table 8).

## 4. Discussion

The modern management of haemophilia has evidently influenced not only clinical symptoms, that is, orthopedic outcome and survival of patients, but also their perceived HRQoL. Lately, QoL measures have become an indispensable part of clinical trials as one of the most essential patient-rated outcomes (PROs). Similarly, QoL assessment has become also important in pharmacoeconomics [22, 23].

Currently, disease-specific questionnaires for assessing patients' HRQoL are increasingly being utilized in routine health monitoring of haemophilia patients. Thus far, there is only one study in Greece measuring HRQoL in haemophilia patients [30]. Nevertheless, in that study, HRQoL of 78 Greek haemophilia patients was assessed using only the generic instruments SF-36 and EQ-5D. Haem-A-QoL is a commonly used disease-specific instrument with very good psychometric characteristics, including good reliability values (Cronbach's *α* ranging between 0.74 and 0.88) and high convergent and discriminant validity [18, 23] and it has been translated into more than 30 languages [20, 21]. Two recent studies, in Turkey and Brazil, use translated versions of these instruments to evaluate health-related quality of life in the corresponding haemophilia populations [31, 32]. However, to the extent of our knowledge, we are unaware of any published validation studies regarding the Greek version of Haem-A-QoL. The present study aimed to assess the reliability and validity of the Greek version of the Haem-A-QoL questionnaire and increase confidence in results from future studies in Greece using this instrument in adult haemophiliacs.

The overall results for the psychometric properties of the instrument were quite satisfactory. In general, no floor and ceiling effects were observed, suggesting good discriminative ability and good responsiveness (Table 2). The single presence of a marginally high floor effect of one Haem-A-QoL scale (relationship/partnership) does not in the least derogate from the acceptable limits for a satisfactory discriminative ability of the Greek version of this instrument.

Scale internal consistency reliability, as expressed by Cronbach's *α* coefficient, was very high in all dimensions, ranging between 0.765 and 0.954 (Table 2), and exceeded the corresponding reliability coefficients reported in recent similar studies [31, 32]. This difference might be attributed to the small sample size of these studies (*n* = 30 and *n* = 39, resp.) compared to our sample size (*n* = 118). Only one dimension (dealing) exhibited in our study borderline acceptable reliability (0.585). The two previously mentioned studies using Haem-A-QoL had similar or even worse Cronbach's *α* (0.6 and 0.13, resp.) in the same Haem-A-QoL dimension [31, 32]. Test-retest reliability coefficients, measured in 19.5% of the sample, were likewise considerably high, ranging from 0.872 to 0.974 (Table 2), implying that scale scores were consistent over time from one test administration to the next and that there was little or no influence from external factors.

The hypothesized structure of the Haem-A-QoL was confirmed with evidence provided by item-internal consistency and item-discriminant validity (Table 3). Both were satisfactory, suggesting that the translation of the items and the response choices are appropriate and that scale scores derived from the Greek version could contribute to cross-cultural comparisons. Criterion-related construct validity was supported by corroboration of hypothesized strong correlations between Haem-A-QoL and SF-36 scales measuring similar dimensions (Table 4).

Multiple stepwise linear regression models exhibited relatively high proportions of HRQoL variance explained. Except for one scale (dealing), all other Haem-A-QoL scales were significant predictors of at least one SF-36 scale, with the dimension “future” contributing to seven and “feelings” to three (Table 5). These findings substantiate that the Greek version of the instrument is indeed measuring standard quality of life aspects.

Known-group comparisons using various demographic and clinical characteristics generated expected results with reference to score variations and the direction of these differences, several of which were also statistically significant, and others were only marginally insignificant. More specifically, age was a significant factor with a negative relationship to perceived health in most Haem-A-QoL scales (Table 6), in accordance with results from adult patients reported elsewhere [5, 6, 30, 33]. Other sociodemographic factors, like educational level, employment, and family status, proved to be significant predictors for certain Haem-A-QoL subscales, influencing negatively patients' quality of life. Specifically, low educational level was associated in a statistically significant manner with deteriorations in four Haem-A-QoL subscales (physical health, feelings, view, and work/school), as well as in total score (Table 6). Similar findings were reported by another study using Haem-A-QoL [19], where patients with low educational level showed significantly more impairment in QoL in the dimensions of feelings, view, treatment, future, and total score. In another study, it is reported that educational level was correlated with the anxiety scale of EQ-5D, but no other correlation was identified with either EQ-5D or SF-36 [30]. The above-mentioned demographic features, age and educational level, were also amongst the most pronounced and significant predictors for most of the Haem-A-QoL dimensions, when multiple OLS regression models were performed. Similarly, family status and employment were strongly associated with two (physical health and family planning) and three (physical health, work/school, and total score) Haem-A-QoL scales, respectively (Table 6).

Among the clinical features, severity of the disease, presence of hematoma, and prophylactic treatment were found to have statistically significant correlations with one, two, and four Haem-A-QoL dimensions, respectively (Table 6). More specifically, in our study patients with severe haemophilia had worse quality of life, compared with those suffering from moderate or mild haemophilia. Only the dimension “sports/leisure” was found to be affected by disease severity in a statistically significant manner, which is consistent with another study [32]. However, results concerning severity are contradictory in the literature. Several studies report that severe haemophilia is associated with worse quality of life scores in certain QoL subscales [5, 12, 34–36]. Elsewhere, it is reported that patients with moderate disease demonstrate lower scores in similar or different quality of life subscales than patients with severe haemophilia [6, 36]. The explanation for this discrepancy given by some authors is that there is a patient selection, in which subjects with moderate disease were more compromised in some respects than those with severe disease [6]. Another possible explanation might be the fact that patients with severe disease might adhere better to treatment therapies in order to overcome their symptoms, whereas patients with moderate or mild haemophilia often exhibit worse treatment adherence and therefore more symptoms.

Prophylaxis, although more expensive than on demand therapy, is currently the preferred treatment strategy, since patients on prophylaxis have less bleeds, are absent fewer days from school or work, spend fewer days in hospitals, and have generally higher QoL. In the present study, only a small percentage (14.4%) was on constant prophylactic treatment; 9.3% were on transient prophylaxis; and the majority of the patients (76.3%) had no prophylaxis. Patients with no prophylaxis showed higher mean scores (worse quality of life) in the majority of the Haem-A-QoL dimensions, with the most statistically significant ones being physical health, work/school, dealing, and relationship/partnership, compared to patients with transient or constant prophylactic treatment. Similar results were reported by various studies in adults and children [12, 36–39]. Contradictory to this, a study from von Mackensen and Gringeri in Italy reported that patients on prophylactic treatment had worse quality of life compared to those on demand strategy. According to the authors, these conflicting findings could be attributed to the fact that patients in Italy receive prophylactic therapy when they are already in a very severe condition [23].

Viral infections are a common clinical feature amongst haemophilia patients, due to old treatment strategies that employed transfusion of blood products. A high percentage of our sample patients were either HCV or HIV infected (71.2% and 28.0%, resp.). Hepatitis B virus was present only in two patients of our sample (1.7%); therefore, its presence was not further studied with regard to HRQoL. As expected, the presence of a viral infection (HCV or HIV) is associated with a continuous decline in self-perceived health in all but one (dealing) Haem-A-QoL dimension (Table 7). Viral infections were identified as significant predictors for seven out of ten Haem-A-QoL subscales and for total score in the regression models (Table 8). Our findings are in accordance with results from numerous previous studies, having reported a negative effect of the presence of either HCV or HIV. Trippoli et al. published substantial worsening of certain QoL subscales, when haemophilia patients had viral infections [6]. Rentz et al, using a patient-reported symptom assessment tool, demonstrated that HIV positive haemophilia patients had more severe symptoms [12]. Lindvall et al. also found that HCV positive haemophilia patients exhibited worse QoL, whereas no differences were identified in HIV positive haemophilia patients [36].

Significant differences in perceived health between the two different types of haemophilia were evident. Patients with haemophilia A exhibited lower mean scores in all Haem-A-QoL dimensions compared with patients with haemophilia B. Differences in health were statistically significant in six subscales and in the total score; however, a trend to significance (*P* = 0.066–0.084) could also be detected to the other subscales (Table 7). This finding is consistent with recent studies, in which higher bleeding frequency has been observed in patients with haemophilia A compared to patients with haemophilia B [40, 41].

Although haemophilia is a rare condition affecting a small percentage of the Greek population (approximately 0.008%), costs are disproportionately high and therefore are of elevated economic interest to health care payers. The high cost of recombinant coagulation factors, combined with the fact that haemophilia is often correlated with considerable morbidity and lifelong treatment of the patients, makes this disease a potential target for cost-reducing efforts by health care providers [42, 43]. Hence, QoL assessments could become an integral part of clinical trials, laying the foundation for future economic evaluations in haemophilia, such as cost-utility and cost-effectiveness.

### 4.1. Limitations of the Study

The study was conducted in only one of the four National Reference Centers of Congenital Bleeding Disorders based in Athens, and one could argue that this restricts the reliability and generalization of results. However, as shown in Table 1, patients visiting this centre come not only from the greater area of Athens (Attica 57.6%), but also from all over Greece (rest of Greece, 42.4%), covering a wider geographical distribution and thus compensating for the restriction of the single center. The small sample size (*n* = 118) could explain some of the discrepancies observed during the statistical analysis. Nonetheless, according to the latest national register of haemophilia patients in Greece, it is estimated that approximately 716 adult patients suffer from either haemophilia A or B. Consequently, our sample represents about 16.5% of the total adult haemophilia patient population, which is adequate enough for a representative study.

## 5. Conclusion

Overall, our findings confirm that the Greek version of Haem-A-QoL is a reasonably good option for measuring health-related quality of life in Greece, filling a current void, and thus contributing to the international literature on the subject. The instrument was well accepted by the patients and proved a reliable and valid tool with psychometric properties similar to those reported in validation studies of the original version and other language versions. The Greek version of Haem-A-QoL can be implemented in the routine assessment of Greek adult patients with haemophilia, since the continuous monitoring of HRQoL parameters can potentially identify specific health care needs of individual patients and verify treatment strategies.

## Figures and Tables

**Table:** (a)

Sociodemographic Data	*n*	%
Age group		
18–30 yrs	33	28.0
31–40 yrs	31	**26.3**
41–50 yrs	30	**25.4**
51–60 yrs	16	13.6
>60 yrs	8	6.8
Gender		
Male	117	**99.2**
Female	1	0.8
Family status		
Unmarried	72	**61.0**
Married	43	36.4
Divorced/widowed/separated	3	2.6
Residence		
Attica	68	**57.6**
Rest of Greece	50	42.4
Educational level		
Elementary	6	5.1
High school	68	**57.6**
Graduate	40	33.9
Postgraduate	4	3.4
Employment		
Unemployed	18	15.3
Employee	38	**32.2**
pensioner	32	27.1
Student	18	15.3
Farmer	4	3.4
Self-employed	8	6.8

**Table:** (b)

Clinical data	*n*	%
Haemophilia		
*Α* (factor V*ΙΙΙ* deficiency)	92	**78.0**
*Β* (factor IX deficiency)	26	22.0
Severity		
Severe	75	**63.6**
Moderate	16	13.5
Mild	27	22.9
Inhibitor		
Yes	9	7.6
No	109	**92.4**
Prophylaxis		
No	90	**76.3**
Transient	11	9.3
Constant	17	14.4
Haemarthrosis		
Yes	92	**78.0**
No	26	22.0
Muscle hematomas		
Yes	106	**89.8**
No	12	10.2
Other bleeding		
Yes	88	**74.6**
No	30	25.4
Factor concentrates		
Recombinant	112	**94.9**
Plasma derived	6	5.1
HIV infection		
Yes	33	28.0
No	85	**72.0**
HCV infection		
Yes	84	**71.2**
No	34	28.8
HBV infection		
Yes	2	1.7
No	116	**98.3**

Bold numbers indicate the demographic and clinical data with the higher percentage.

HIV: human immunodeficiency virus, HCV: hepatitis C virus, and HBV: hepatitis B virus.

**Table 2:** Central tendency, variability, and reliability of the Haem-A-QoL scales.

	Number of items	Mean (SD)	95% CI	Median	Floor (%)	Ceiling (%)	Cronbach's *α*	Test-retest reliability
Physical health	5	42.97 (23.44)	38.69–47.24	45.00	0 (7.6)	95 (0.8)	0.871	0.876
Feelings	4	35.33 (23.49)	31.05–39.61	31.25	0 (12.7)	100 (0.8)	0.863	0.974
View	5	40.93 (20.64)	37.17–44.69	40.00	0 (0.8)	100 (0.8)	0.774	0.960
Sports/leisure	5	61.06 (21.81)	57.08–65.03	65.00	0 (1.7)	100 (1.7)	0.810	0.963
Work/school	4	31.41 (19.77)	27.80–35.01	31.25	0 (11)	81.3 (0.8)	0.808	0.950
Dealing	3	27.05 (19.49)	23.49–30.60	25.00	0 (9.3)	100 (1.7)	0.585	0.872
Treatment	8	31.25 (17.32)	28.09–34.41	28.12	0 (0.8)	84.4 (0.8)	0.765	0.949
Future	5	39.15 (20.31)	35.45–42.86	40.00	0 (2.5)	95 (0.8)	0.806	0.965
Family planning	4	25.85 (23.34)	21.59–30.10	25.00	0 (15.3)	100 (2.5)	0.876	0.910
Relationship/partnership	3	24.51 (30.14)	19.01–30.00	8.33	0 (40.7)	100 (2.5)	0.954	0.981
Total Haem-A-QoL	46	33.59 (14.53)	30.94–36.23	33.97	4.9 (0.8)	71.7 (0.8)	0.872	—

SD: standard deviation; 95% CI: 95% confidence interval.

**Table 3:** Summary results of scaling assumptions tests.

	N*	Item-internal consistency		Item-discriminant validity	
		Range of correlations^†^	Success/total^‡^	Range of correlations^§^	Success/total^¶^
Physical health	5	0.624–0.766	5/5	0.08–0.775	37/45
Feelings	4	0.670–0.740	4/4	0.03–0.779	28/36
View	5	0.362–0.661	4/5	0.12–0.770	40/45
Sports/leisure	5	0.530–0.727	5/5	0.01–0.780	38/45
Work/school	4	0.598–0.672	4/4	0.06–0.772	31/36
Dealing	3	0.229–0.529	2/3	0.49–0.787	27/27
Treatment	8	0.045–0.668	5/8	0.04–0.772	62/72
Future	5	0.309–0.758	4/5	0.09–0.729	36/45
Family planning	4	0.563–0.827	4/4	0.13–0.775	29/36
Relationship/partnership	3	0.897–0.909	3/3	0.22–0.797	13/36

*Number of questions and number of item-internal consistency tests per dimension.

^†^Range of correlations between questions and their corresponding dimension.

^‡^Number of correlations that are over the acceptable limit of 0.40/total number of correlations.

^§^Range of correlations between questions and other dimensions.

^¶^Number of successful tests of item-discriminant validity/total number of item-discriminant validity tests.

**Table 4:** Spearman's rank correlations between Haem-A-QoL and SF-36 scales.

Haem-A-QoL scales	SF-36 scales									
	PF	RP	BP	GH	VT	SF	RE	MH	PCS	MCS
Physical health	−***0.66*** ^∗∗∗#^	***−0.58*** ^∗∗∗#^	***−0.68*** ^∗∗∗#^	−0.56***	−0.51***	−0.57***	−0.33***	−0.45***	***−0.75*** ^∗∗∗#^	−0.33***
Feelings	−0.49***	−0.56***	−0.57***	−0.62***	−0.66***	−0.60***	−0.43^∗∗∗#^	***−0.55*** ^∗∗∗#^	−0.60***	***−0.53*** ^∗∗∗#^
View	−0.46***	−0.56***	−0.51***	−0.69***	−0.61***	−0.55***	−0.42***	−0.57***	−0.58***	−0.52***
Sports/leisure	−0.60***	−0.49***	−0.55***	−0.61***	−0.46***	−0.58***	−0.40***	−0.36***	−0.64***	−0.39***
Work/school	−0.56***	−0.62***	−0.56***	−0.58***	−0.49***	−0.54***	−0.44***	−0.44***	−0.67***	−0.40***
Dealing	−0.08	−0.07	−0.13	−0.18*	−0.12	−0.06	−0.06	−0.12	−0.10	−0.08
Treatment	−0.53***	−0.62***	−0.56***	−0.61***	−0.54***	−0.53***	−0.40***	−0.52***	−0.65***	−0.43***
Future	−0.55***	−0.66***	−0.71***	−0.75***	−0.70***	−0.67***	−0.47***	−0.68***	−0.71***	−0.59***
Family planning	−0.25**	−0.28**	−0.30***	−0.42***	−0.35***	*−0.47* ^∗∗∗#^	−0.26***	−0.33***	−0.32***	−0.37***
Relationship/partnership	−0.46***	−0.35***	−0.30***	−0.49***	−0.43***	*−0.45* ^∗∗∗#^	−0.38***	−0.41	−0.41***	−0.41***

*Notes*. ^#^Numbers in italics indicate correlations hypothesized to be moderate or strong. Negative correlations are an artifact of the scoring procedures.

PF: physical functioning, RP: role physical, BP: bodily pain, GH: general health, VT: vitality, SF: social functioning, RE: role emotional, MH: mental health, PCS: physical component summary score, and MCS: mental component summary score.

*P < 0.05; **P < 0.01; ***P < 0.001, with Bonferroni adjustment for multiple testing.

**Table 5:** Multivariate analysis (*B*  coefficient^(*P*-sig.)^).

Dependent variable predictors	Dependent variable							
	PF	RP	BP	GH	VT	SF	RE	MH
Physical health	−0.54***	—	−0.41***	—	—	—	—	—
Feelings	—	—	—	—	−0.51***	−0.24**	—	−0.51**
View	—	—	—	−0.28***	—	—	—	—
Sports/leisure	—	—	—	−0.25***	—	−0.48***	—	—
Work/school	—	−0.69***	—	—	—	—	—	—
Dealing	—	—	—	—	—	—	—	—
Treatment	−0.31*	−0.70***	—	—	—	—	—	—
Future	—	−0.66***	−0.57***	−0.49***	−0.32***	−0.28**	−0.713***	−0.19***
Family planning	—	0.30*	—	—	0.14*	—	—	—
Relationship/partnership	−0.14*	—	—	—	—	—	−0.302**	—
*Constant *	102.60***	110.25***	94.0***	91.78***	86.37***	110.24***	102.86***	91.58***
*Adjusted R* ^2^	*0.491 *	*0.536 *	*0.583 *	*0.646 *	*0.579 *	*0.525 *	*0.246 *	*0.466 *

PF: physical functioning, RP: role physical, BP: bodily pain, GH: general health, VT: vitality, SF: social functioning, RE: role emotional, and MH: mental health.

*P < 0.05; **P < 0.01; ***P < 0.001.

**Table 6:** Statistical significant correlations between Haem-A-QoL scales and sociodemographic and clinical characteristics.

Haem-A-QoL dimensions	*P*value									
	Age group	Family status	Employment	Education level	Haem A/B	Severity	Hematomas	Prophylaxis	HCV infection	HIV infection
Physical health^a^	0.002	0.023	<0.001	0.013				0.022	<0.001	0.026
Feelings^a^				0.007	0.015					
View^a^	0.020			0.009					0.041	0.009
Sports/leisure^b^	0.022				0.006	0.018			0.001	<0.001
Work/school^a^	0.024		0.040	<0.001	0.014			0.007	0.002	0.002
Dealing^b^								0.057		
Treatment^a^	0.021				0.008		0.023			
Future^a^									0.001	0.031
Family planning^b^		0.048							0.001	0.002
Relations/partnership^b^	0.025				0.001			0.038		0.001
Total Haem-A-QoL^a^	0.003		0.009	0.002			0.199		0.001	0.001

Haem A/B stands for haemophilia type A or B.

^a^Analysis was performed using independent samples *t*-test or ANOVA. ^b^Analysis was performed using Mann-Whitney test or Kruskal-Wallis test.

**Table 7:** Stratified Haem-A-QoL scores according to age and health-related characteristics of the sample.

	*n*		PH^a^	FL^a^	VW^a^	SL^b^	WS^a^	DL^b^	TR^a^	FU^a^	FP^b^	RP^b^	THQ^a^
Age group													
18–30 years	33		30.91	29.26	33.64	52.88	23.48	21.71	25.00	31.52	17.05	13.89	26.27
31–40 years	31		41.13	31.65	38.06	58.39	30.65	27.15	29.23	40.32	33.06	24.19	32.84
41–50 years	30		50.00	40.42	47.33	68.17	35.63	28.06	37.92	44.00	27.92	34.17	38.79
51–60 years	16		51.88	39.06	42.50	66.56	34.37	31.25	31.05	42.50	30.47	27.08	35.55
>60 years	8		55.63	47.66	55.00	67.50	45.31	36.46	40.23	41.25	17.19	28.13	41.24
		*P* (sig.)	**0.002**	0.139	**0.020**	**0.022**	**0.024**	0.533	**0.021**	0.134	0.125	**0.025**	**0.003**
Haemophilia type													
A	92		45.00	38.11	42.61	63.70	33.76	27.81	33.49	40.98	27.92	28.89	35.67
B	26		35.77	25.48	35.00	51.73	23.08	24.36	23.32	32.69	18.51	8.97	26.21
		*P* (sig.)	0.076	**0.015**	**0.097**	**0.006**	**0.014**	0.084	**0.008**	0.066	0.079	**0.001**	**0.001**
HIV infection													
Yes	33		49.70	41.29	48.79	72.27	40.15	25.25	34.47	45.61	37.12	37.88	40.00
No	85		40.35	33.01	37.88	56.71	28.01	27.75	30.00	36.65	21.47	19.31	31.10
		*P* (sig.)	**0.026**	0.086	**0.009**	**<0.001**	**0.002**	0.850	0.210	**0.031**	**0.002**	**0.001**	**0.001**
HCV infection													
Yes	84		48.63	37.50	43.39	65.06	34.90	26.29	33.11	43.10	30.21	27.28	36.45
No	34		28.97	29.96	34.85	51.18	22.79	28.92	26.65	29.41	15.07	17.65	26.52
		*P* (sig.)	**0.001**	0.115	**0.041**	**0.001**	**0.002**	0.731	0.066	**0.001**	**0.001**	0.066	**0.001**

PH: physical health, FL: feelings, VW: view, SL: sports/leisure, WS: work/school, DL: dealing, TR: treatment, FU: future, FP: family planning, RP: relationship/partnership, and THQ: total haem-A-QoL. ^a^Analysis was performed using independent samples *t*-test or ANOVA. ^b^Analysis was performed using Mann-Whitney test or Kruskal-Wallis test.

**Table 8:** Multiple stepwise linear regression models.

	Predictors	*B *	SE	*t*	*P* value	Adjusted *R* ^2^
Physical health	(Constant)	58.05	3.991	14.546	**<0.001**	**0.146**
	HCV	−9.61	2.181	−4.407	**<0.001**	
Feelings	(Constant)	49.10	6.696	7.333	**<0.001**	**0.037**
	Haemophilia type	−11.68	5.154	−2.266	**0.025**	
View	(Constant)	71.82	8.883	8.085	**<0.001**	**0.090**
	HIV	−12.64	4.305	−2.936	**0.004**	
	Prophylaxis	−6.63	3.066	−2.164	**0.033**	
Sports/leisure	(Constant)	99.65	8.609	11.576	**<0.001**	**0.173**
	HIV	−10.60	4.587	−2.311	**0.023**	
	HCV	−5.32	2.169	−2.451	**0.016**	
	Haemophilia type	−9.50	4.413	−2.153	**0.034**	
Work/school	(Constant)	57.26	8.384	6.830	**<0.001**	**0.200**
	HCV	−5.02	1.913	−2.622	**0.010**	
	Prophylaxis	−9.21	2.629	−3.504	**0.001**	
	HIV	−9.48	4.019	−2.359	**0.020**	
	Bleedings	7.95	3.837	2.073	**0.041**	
Dealing	(Constant)	35.22	4.390	8.023	**<0.001**	**0.032**
	Prophylaxis	−6.50	3.049	−2.131	**0.035**	
Treatment	(Constant)	26.51	8.239	3.217	**0.002**	**0.083**
	Haemophilia type	−8.66	3.693	−2.345	**0.021**	
	Hematomas	13.88	5.953	2.332	**0.022**	
Future	(Constant)	44.33	8.660	5.119	**<0.001**	**0.155**
	HCV	−6.84	1.906	−3.586	**0.001**	
	Prophylaxis	−7.72	2.841	−2.718	**0.008**	
	Hematomas	14.38	6.658	2.160	**0.033**	
Family status	(Constant)	54.91	8.504	6.456	**<0.001**	**0.099**
	HIV	−16.96	4.732	−3.584	**0.001**	
Relationships/partnership	(Constant)	78.54	13.191	5.954	**<0.001**	**0.132**
	Haemophilia type	−13.44	6.335	−2.121	**0.036**	
	HIV	−14.6	6.028	−2.422	**0.017**	
	Prophylaxis	−10.05	4.284	−2.346	**0.021**	
Total Haem-A-QoL	(Constant)	55.62	4.692	11.854	**<0.001**	**0.181**
	HCV	−4.88	1.307	−3.737	**<0.001**	
	Haemophilia type	−7.42	2.856	−2.598	**0.011**	
	Prophylaxis	−4.23	1.964	−2.155	**0.033**	

Physical health	(Constant)	26.31	4.608	5.710	**<0.001**	**0.118**
	Age group	6.81	1.678	4.058	**<0.001**	
Feelings	(Constant)	62.29	8.023	7.764	**<0.001**	**0.086**
	Educational level	−11.37	3.285	−3.461	**0.001**	
View	(Constant)	63.55	7.088	8.966	**<0.001**	**0.078**
	Educational level	−9.55	2.902	−3.290	**0.001**	
Sports/leisure	(Constant)	85.09	7.477	11.380	**<0.001**	**0.079**
	Educational level	−10.13	3.061	−3.309	**0.001**	
Work/school	(Constant)	63.50	6.392	9.935	**<0.001**	**0.182**
	Educational level	−13.56	2.617	−5.181	**0.001**	
Dealing	(Constant)	19.05	4.017	4.743	**<0.001**	**0.033**
	Age group	3.26	1.463	2.225	**0.028**	
Treatment	(Constant)	22.95	3.526	6.510	**<0.001**	**0.050**
	Age group	3.43	1.284	2.667	**0.009**	
Future	(Constant)	54.71	7.123	7.681	**<0.001**	**0.033**
	Educational level	−6.53	2.916	−2.238	**0.027**	
Total Haem-A-QoL	(Constant)	40.23	6.813	5.905	**<0.001**	**0.135**
	Educational level	−5.51	2.156	−2.553	**0.012**	
	Age group	2.63	1.123	2.338	**0.021**	

*B*: factor dependence.

SE: standard error.
